# Unintended consequences of patient online access to health records: a qualitative study in UK primary care

**DOI:** 10.3399/BJGP.2021.0720

**Published:** 2022-11-01

**Authors:** Andrew Turner, Rebecca Morris, Lorraine McDonagh, Fiona Hamilton, Sarah Blake, Michelle Farr, Fiona Stevenson, Jon Banks, Helen Atherton, Dylan Rakhra, Gemma Lasseter, Gene Feder, Sue Ziebland, Emma Hyde, John Powell, Jeremy Horwood

**Affiliations:** National Institute for Health and Care Research Applied Research Collaboration West (NIHR ARC West), University Hospitals Bristol and Weston NHS Foundation Trust, Bristol; Centre for Academic Primary Care (CAPC), University of Bristol, Bristol Medical School, Bristol.; NIHR Greater Manchester Patient Safety Translational Research Centre, Centre for Primary Care, University of Manchester, Manchester.; Research Department of Primary Care and Population Health, University College London, London.; Research Department of Primary Care and Population Health, University College London, London.; University of Bristol, Bristol.; National Institute for Health and Care Research Applied Research Collaboration West (NIHR ARC West), University Hospitals Bristol and Weston NHS Foundation Trust, Bristol; Centre for Academic Primary Care (CAPC), University of Bristol, Bristol Medical School, Bristol.; Research Department of Primary Care and Population Health, University College London, London.; National Institute for Health and Care Research Applied Research Collaboration West (NIHR ARC West), University Hospitals Bristol and Weston NHS Foundation Trust, Bristol; Centre for Academic Primary Care (CAPC), University of Bristol, Bristol Medical School, Bristol.; Unit of Academic Primary Care, University of Warwick, Coventry.; Department of Philosophy/ School of Oral and Dental Sciences, University of Bristol, Bristol.; Health Protection Research Unit in Behavioural Science and Evaluation, University of Bristol, Bristol.; National Institute for Health and Care Research Applied Research Collaboration West (NIHR ARC West), University Hospitals Bristol and Weston NHS Foundation Trust, Bristol; Centre for Academic Primary Care (CAPC), University of Bristol, Bristol Medical School, Bristol.; Nuffield Department of Primary Care Health Sciences, University of Oxford, Oxford.; School of Sociology and Social Policy, University of Leeds, Leeds.; Nuffield Department of Primary Care Health Sciences, University of Oxford, Oxford.; National Institute for Health and Care Research Applied Research Collaboration West (NIHR ARC West), University Hospitals Bristol and Weston NHS Foundation Trust, Bristol; Centre for Academic Primary Care (CAPC), University of Bristol, Bristol Medical School, Bristol.

**Keywords:** digital first primary care, digital health, electronic health records, general practice, primary health care, qualitative interviews

## Abstract

**Background:**

Health systems are seeking to harness digital tools to promote patient autonomy and increase the efficiency of care worldwide. The NHS Long Term Plan created the right for patients to access ‘digital first’ primary care by 2023–2024, including online patient access to full medical records.

**Aim:**

To identify and understand the unintended consequences of online patient access to medical records.

**Design and setting:**

Qualitative interview study in 10 general practices in South West and North West England.

**Method:**

Semi-structured individual interviews with 13 patients and 16 general practice staff with experience of patient online access to health records.

**Results:**

Online access generated unintended consequences that negatively impacted patients’ understanding of their health care, with patients finding surprising or difficult to interpret information. Online access impacted GPs’ documentation practices, such as when GPs pre-emptively attempted to minimise potential misunderstandings to aid patient understanding of their health care. In other cases, this negatively impacted the quality of the records and patient safety when GPs avoided documenting speculations or concerns. Contrary to assumptions that workload would be reduced, online access introduced extra work, such as managing and monitoring access, and taking measures to prevent possible harm to patients.

**Conclusion:**

The unintended consequences described by both staff and patients show that, to achieve the intended consequences set out in NHS policy, additional work is necessary to prepare records for sharing and to prepare patients about what to expect. It is crucial that practices are adequately supported and resourced to manage the unintended consequences of online access, now that it is the default position. A table of potential unintended consequences and mitigation measures is provided to aid practice managers and clinicians implementing online access.

## INTRODUCTION

Political, economic, and social pressures are driving patient access to health records globally.^1^^–^4 Healthcare systems in many countries make medical records accessible to patients in some form, although there is considerable variability in what and how information is available to patients.^5^

Online patient access to their medical records (henceforth ‘online access’) satisfies the moral argument that the information rightly belongs to the patient and so should be accessible to them.^4^^,^^6^ More instrumentally, online access is intended to enable patients to take greater control of their health, in parallel with increasing the efficiency of care and delivering reductions in GP practice workload.^7^^–^12 However, these are relatively new roles for medical records to perform, and are in contrast to their traditional roles of supporting clinicians in the care of patients and providing evidence for legal matters, audit, and research.^13^

In the UK, online access to summary information in primary care records has been available to patients since 2015, and, before this, the right to a copy of one’s medical record (typically a print copy) was part of data protection legislation. The NHS Long Term Plan created the right for patients to access ‘digital first’ primary care by 2023–2024.^10^ Although the rollout of online record access has been delayed, from November 2022 users of the NHS app (or similar apps) should have full record access prospectively, and retrospective access to their coded records in 2023.^14^

NHS England patients can be given online access by registering for online services at their GP practice. (Online services also include other linked services, such as booking appointments and ordering repeat prescriptions, which are not the subject of this study.) Patients can access their records online through various providers.^15^ Access is available at two levels: a ‘detailed coded record’ (DCR) and a ‘full record’. DCRs contain information about a patient’s allergies, immunisations, medications, and test results, as well as coded problems, diagnoses, and procedures, although what is made available from this list varies between practices. Full records contain the DCR, plus free-text clinician consultation notes and other documents such as hospital letters. However, results from recent GP Patient Surveys show that only 7% of patients said they were using online services to access their medical records.^16^^,^^17^

**Table:** How this fits in

Previous studies of patient online access to medical records have noted a range of concerns about potential unintended consequences. This study reports real-world experiences of the consequences of online access. Unintended consequences were identified that impacted patient autonomy and GP documentation practices, and also increased workload through providing access while avoiding harm to patients. It is crucial that practices are adequately supported and resourced to manage the unintended consequences of online access, now that it is the default position.

Studies of online access to medical records have found some evidence that it can improve health outcomes and patient safety.^6^^,^^18^ Studies have also found that patients have used online access to better understand and control their health,^19^ for example, by allowing them to better prepare for subsequent consultations and correct errors,^18^^,^^20^^–^24 while finding the technology *‘convenient, useful, usable, and flexible’*.^25^

Evaluations of digital health tools have found that their promise is not always delivered, however, and real-world implementation frequently produces unintended consequences.^26^^–^28 These are positive or negative effects that were not intended at the outset, but which often occur when adopting novel technologies.^29^ Possible unintended consequences of online access include concerns about confidentiality and risk of patient coercion,^3^^,^^30^ patient confusion and anxiety,^3^^,^^6^^,^^31^ the creation of additional clinician workload,^19^ and widening health inequalities.^32^^,^^33^ However, these are often speculative and hypothetical concerns, with unclear evidence on how they are realised in practice.^3^

The aim of this study was to identify and understand the unintended consequences of online access to health records experienced by patients and practices, to inform guidance on mitigation of these consequences at practice and policy levels.

## METHOD

Semi-structured individual interviews were conducted with patients and staff from GP practices in South West and North West England in 2019 and 2020. This article reports results from the DECODE study,^34^ which examined the unintended consequences of three types of digital health tool in primary care: online consultation tools, patient online access to health records, and smartphone apps to help patients manage long-term conditions. Only results about online access to health records are reported here. Results about online consultations are published elsewhere.^35^

Unintended consequences were defined in contrast to the intended consequences set out in policy documents. NHS England policy documents suggest that there are two main intended consequences of providing streamlined access to information about one’s health through online access to health records:^7^^–^10^,^^12^

### Intended consequence 1: Enable patients to take greater control of their health

Providing *‘information about their condition and history’* to support more *‘personalised care* … *wellbeing and independence’* ,^8^ helping patients *‘manage* [their] *own health and care better’* and enabling *‘more informed discussions and genuine involvement in decisions about* [their] *health and care’*.^7^

### Intended consequence 2: Improve the efficiency of care or improve practice workload

Creating *‘significant increases in productivity that far outweigh the initial investment’* through, for example, *‘reductions in* ad hoc *contacts with some patients’*^7^ and enabling ‘ *care to be designed and delivered in the place that is most appropriate for* [patients, clinicians, and their carers] *’*.^10^

In this study, consequences are labelled as unintended if they did not fall under the intended consequences given above. Unintended consequences could be positive or negative, or could be anticipated or not.

### Sampling and recruitment

Research-active practices in eight clinical commissioning group (CCG) areas in the South West and North West of England were provided with study information by the National Institute for Health and Care Research Clinical Research Network. Expressions of interest were received and practices were selected according to their level of experience with the three types of digital health tool being investigated in the DECODE study. Where possible, practices were selected to provide a mix in relation to size, urban/rural location, and indices of area-level socioeconomic scores for the practice population,^36^ although these characteristics are not evenly represented in the final sample (Table 1).

**Table 1.** Practice characteristics

**Site**	**Patient list size^a^**	**IMD quintile^b^**	**Location**	**Level of patient online access to health records^c^**	**Staff interviewed, *n***	**Patients interviewed, *n***
**1**	Medium	5	Urban	DCR	1	1
**2**	Small	5	Urban	DCR	2	5
**3**	Medium	5	Rural	DCR	2	0
**4**	Large	2	Urban	Full record	4	2
**5**	Medium	5	Urban	DCR	0	2
**6**	Small	5	Rural	Full record	0	1
**7**	Small	5	Urban	DCR	1	0
**8**	Large	2	Urban	DCR	2	0
**9**	Medium	2	Urban	Full record	1	0
**10**	Small	4	Urban	DCR	3	2

a *Small <10 000; medium 10 000–14 999; large ≥15 000.*

b *1 = more deprived; 5 = less deprived (based on practice postcode).*

c *DCR. DCR = detailed coded record. IMD = Index of Multiple Deprivation.*

Practice staff were recruited through the practice manager or research lead. Patients were eligible to take part if they were registered for online services, or were known to staff as having requested full-record access. When large numbers of patients met these criteria, the set of those invited was targeted by age, ethnicity, and long-term condition to try to maximise diversity. Eligible patients were sent invitation letters by participating practices or were opportunistically provided with study information by clinical staff.

The concept of ‘information power’^37^ informed analysis, sampling, and participant recruitment, which were conducted in parallel to allow sampling to be refined as the project developed. Information power is a guiding principle in qualitative research, suggesting that studies with broad aims and exploratory analysis may need larger samples, while smaller samples can be sufficient if data are focused and if participants have rich experiences relevant to the research question.

### Data collection

Topic guides were developed by the study team and informed by a stakeholder workshop held in 2018 to explore possible unintended consequences of digital health technology.^34^^,^^38^ Topic guides were refined iteratively as interviews and preliminary analysis progressed (see Supplementary Appendix S1 for the final version). Interviews were conducted between February 2019 and January 2020 by two authors (face-to-face or by telephone) and lasted 20–60 min (mean 38 min).

### Analysis

Interviews were fully transcribed and coded using QSR NVivo (version 12) software. Thematic analysis^39^ was used to explore staff and patients’ descriptions of the consequences of online record access. Initial noting of ideas was followed by line-by-line examination and inductive coding. The first three transcripts were coded independently and discrepancies discussed to contribute to the generation and refinement of codes to maximise rigour. The coding frame was further refined through discussion with the whole study team, including public and patient involvement (PPI) contributors. Themes were examined to determine whether the consequences being described were in line with the intended consequences outlined above, or were unintended consequences.

## RESULTS

### Practice and participant characteristics

Interviews were conducted before April 2020, so practices that participated were not yet obliged to offer full-record access to existing patients. Characteristics of the 10 practices are shown in Table 1 and 29 participants interviewed in Table 2.

**Table 2.** Participant characteristics

**Characteristics of patients (*N* = 13)**	***n***

Sex	
Female	9
Male	4
Age, years	
30–54	2
55–64	9
≥65	2
Ethnicity	
White British	13

**Characteristics of GP practice staff (*N* = 16)**	***n***

Sex	
Female	8
Male	8
Staff role	
GP	10
Administrative/managerial	6
Average years GP qualified	21

Findings are presented according to the predefined intended consequences (see above), illustrated with anonymised verbatim quotes. Intended consequences are considered first, before unintended consequences.

### Intended consequence 1: Enable patients to take greater control of their health

Staff and patients described online access primarily providing a more convenient way for patients to view information about their health care. Patients reported that this enabled patients to check test results and treatment plans, and to remind themselves about the content of previous consultations. It also occasionally equipped them to challenge their GP or take more control in subsequent consultations. For example, as one patient explained:

*‘My last consultation* […] *I just took him* [the GP] *a list of about fifteen questions about my condition* [… record access has] *certainly helped me to ask more questions or to know a bit more about* [my condition]. ’

(Patient [P]2, Practice [Pr]6)

#### Unintended consequences

Patients and staff highlighted unintended consequences of online access that challenged the intended goal of supporting patient ‘wellbeing and independence’, and enabling their ‘genuine involvement’ in their health and care (see the definitions of intended consequences above). First, some patients described discovering information in their health records that had surprised and distressed them, and which their original consultation had not prepared them for:

*‘I went onto the patient record* […] *to look for if any of the blood test results had come back* [… and it] *said, “urgent referral request: suspected breast cancer”.* […] *You’re instantly like, “Christ! The doctor thinks that I’ve got breast cancer.”’*

(P1, Pr2)

GPs and administrative and managerial staff had little awareness of whether patients experienced this kind of unintended consequence, despite their concern that online access could cause patients distress if they learned something of which they were previously unaware.

Second, patients noted that simple access to information did not necessarily equate to greater involvement or better management of their own health and care. Some patients explained that *‘sometimes too much information can be unhelpful’* (P1, Pr2) or that *‘a little bit of knowledge is a dangerous thing’* (P2, Pr10), especially if they did not have sufficient context to interpret it, and searched online about the *‘disadvantages or drawbacks’* (P2, Pr6) of a condition. One GP described a conversation with a patient who no longer wanted online access because they did not want to read about their growing medical problems:

*‘There were two or three things that* [the patient] *had to remember* [following the consultation] *and so for me it’s always been natural, “just go online and check so you can remind yourself”.* […] *And she was like, “Oh, I don’t really look at my records any more”, I said, “Why not?” “Well, because I don’t want to … I’m scared of seeing something that means I’ve got another problem on top.”’*

(GP1, Pr9)

Third, GPs suggested that information in the record was not tailored to the needs of patients, which could lead to misunderstandings or misinterpretation. For example, GPs noted that patients could struggle to interpret test results, particularly when clinically unimportant information was also visible. GPs thought that their own documentation practices could be a source of difficulty for patients, explaining that consultation notes were written using abbreviations, jargon, and in a time-constrained context where accuracy of spelling was not always prioritised:

*‘There’s a heck of a lot of spelling mistakes we make in our notes.* [A patient] *who requested all her notes, I did have that conversation with her, I said, “There will be lots of spelling mistakes and things” and forewarned her.’*

(GP1, Pr4)

Fourth, online access could have wider medicolegal or patient safety consequences when concern about how information might be interpreted by patients affected the content of consultation notes. For example, some GPs described the difficulty of recording their concerns in notes that would be visible to patients:

*‘*[If] *you’re worried about domestic violence or drug use or something like that and you’re not necessarily firm enough, you might have tried to explore it* [… but the patient is] *coming in and doctor hopping* […] *you can put on a note* [in the record] *to that effect* […] *but you know I’d be very wary of doing that now. I might go and phone the* [other] *doctor myself if I know they* [the patient] *were coming in and have a chat with them* [the doctor]. *But I definitely feel you have to be careful about what you’re putting down in the record* . *’*

(GP1, Pr10)

Notes about possible diagnoses or GP ‘gut feelings’ were described in similar terms: such speculation was thought to leave *‘hostages to fortune’* (GP3, Pr1) if made visible to patients, particularly when the pressures of consultations meant that it was not possible or practical to discuss an issue thoroughly with them. In these cases, speculative but important information was either not documented or shifted to less formal channels.

GPs suggested that mitigating all four kinds of unintended consequence could be achieved through their already cautious and transparent documentation practices. GPs described how their notes were *‘factual’* (GP1, Pr4), *‘objective* [and] *defensive’* (GP2, Pr2) to minimise potential issues for patients viewing them. One GP explained how they write notes without jargon to aid patient understanding:

*‘*[For a female patient] *for instance,* [the] *instructions I wrote was* [ *sic*] *written in a way so I knew if she reads it she will be able to understand, rather than using shorthand* . *’*

(GP1, Pr9)

Another GP described how they had adapted the terminology they use: avoiding *‘normal’* in test results and instead calling statistically abnormal but clinically normal results *‘satisfactory’* (GP1, Pr2) to avoid confusion. GPs also gave examples of writing notes transparently and jointly with patients by explaining to patients what is being documented as they write it:

*‘I do transparent practice, so* [I] *verbalise what I’m finding with patients, I verbalise what I think is going on in my records, I will only put “my impression is this”.* […] *So I’d hope that you’re not going to get things back in your face* . *’*

(GP1, Pr8)

### Intended consequence 2: Improve the efficiency of care or improve practice workload

The primary way that staff found patient online access improved practice workload was by shifting the responsibility for producing copies of medical records. Instead of practices printing out copies (possibly multiple times), staff and patients highlighted the efficiency of allowing patients to extract information themselves as and when they pleased.

#### Unintended consequences: efficiency of care and workload

Patients appreciated the convenience of being able to view their record when they pleased, but beyond this they did not comment on the efficiency of the care they received. Consequently, the unintended consequences below focus on staff experiences of five kinds of addition to practice workload.

First, the preparation of records before giving patients access added to staff workloads. Preparing records included tasks such as redacting sensitive information (that is, information that *‘would be a risk of harm to* […] *the patient or somebody else’* [Administrator (A)1, Pr4]) and references to third parties (such as individuals other than the patient who can be identified from information in the record):

*‘We certainly all anecdotally talk about the work that it’s created with many of the admin staff having to go through and take out third-party references and things. Giving patients ownership over their health can be a good thing, but it can also generate work in other ways.’*

(GP1, Pr4)

Although software was sometimes available to help, this *‘only solve*[d] *part of the problem’* (A1, Pr7) and manual checking was often needed.

Second, online access increased workload when clinical staff provided support to patients requesting access. In these cases, supporting and *‘preparing patients’* (GP1, Pr9) was necessary to help patients know what to expect, and avoid misunderstandings and surprises. Some GPs provided patients with information leaflets or questionnaires, but there were also instances where GPs had arranged a face-to-face consultation with particular patients so they could go through important parts of the record together:

*‘I had a patient who had quite a lot of terrible things* [happen] *to her in her childhood* […] *she wanted online access and so when she requested it* […] *I said, “You are going to have online access. What I think would be good if we go through your record now.”* [The record was] *not that massive and I just went through the problem codes with her so that she could see what they were and that they made sense to her* . *’*

(GP2, Pr4)

Third, additional workload was also generated when managing access to records of teenagers (around 13 years). For example, situations that generated extra work included: practices being asked to redact comments in the child’s record that parents do not want the child to see (or vice versa); allowing access for parents who have separated; and parents requesting access to a child’s record. As well as creating extra administrative work, some situations also meant navigating tricky conversations with parents and children. For example, at one practice where parental access ended when a child turned 13 years of age:

*‘There have been a couple of occasions when that’s actually been really quite difficult, where the kids find themselves in a difficult position of parents saying, “Well why can’t I have access to the notes?” And you say,* “Well it’s set up for your child to say whether you have this or not” and the parents will say, “Well they won’t mind, that will be fine, of course it will”, the child is there and you can see the child squirming […] If you know the situation, you can actually engineer [a way out of] it, but sometimes that can be quite noisy and quite difficult.’

(GP1, Pr2)

Fourth, staff recognised risks around online access for patients experiencing domestic violence and abuse, and described the difficulty of ensuring that access would not cause harm if perpetrators viewed their partner’s record. An unintended consequence of online access, therefore, was the additional work undertaken by staff to minimise harm, such as considering requests on a case-by-case basis and attempting to see the patient alone, face-to-face, to confirm access requests were from them and for them:

*‘* [Domestic violence and abuse is] *one of the reasons why the process* [to get access] *is a bit more long-winded now* [… a consultation in advance allows] *the GP to speak to the patient and that’s kind of why we want the face-to-face because they can be alone in the room* […] *the GP just needs to make sure that they actually want the access themselves and it’s for them.’*

(A1, Pr4)

Fifth, once online access was available, staff described unintended additional workload from managing and monitoring access, such as queries from patients challenging information or finding errors in their record that required correction. Staff noted that genuine errors were typically easy to amend, whereas patient disagreement with otherwise appropriate codes had the unintended consequence of generating difficult discussions around topics such as obesity:

*‘… people don’t like to see things that may be negative about themselves* […] *we do tend to find it’s the obesity stuff that they* [patients] *object to* . *’*

(Practice manager [PM]1, Pr10)

Although online access generated practice workload in a range of ways, staff sometimes down-played the impact of online access because access requests were processed on an *ad hoc* basis, and therefore spread out. Patient uptake was relatively low at the majority of practices:

*‘I didn’t really see that it was going to be that great a change and in our experience it hasn’t been that great a change. It’s still a very low number of patients that want to see their notes and records, or have access to them, in fact we had difficulty convincing patients to sign up* . *’*

(PM1, Pr4)

## DISCUSSION

### Summary

The intended consequences of online access in policy documents are to improve control of one’s health, the efficiency of care, and practice workload. However, the implementation of online access is more complex than the intended consequences would suggest. Online access generated unintended consequences that negatively impacted patients’ control over and understanding of their health, such as when patients discovered surprising information or information was difficult to interpret. Online access impacted GPs’ documentation practices, in some cases potentially aiding patients, such as when GPs pre-emptively attempted to minimise potential misunderstandings. In other cases it negatively impacted the quality of the record when GPs avoided documenting their speculations or concerns, which could have negative medicolegal and patient safety consequences. Contrary to assumptions that practice workload would be reduced, online access introduced extra work, such as managing and monitoring access and taking measures to prevent possible harm to patients.

### Strengths and limitations

A key strength of this research is that it reports the actual experiences of patients and staff using online access, rather than their hypothetical concerns about potential unintended consequences.

Patient experiences of unintended consequences were limited because practices had low numbers of patients using record access. Staff had experienced minor impacts from the increased workload that providing access requires and had minimal experience of patient harm resulting from online access (although some examples of significant, distressing surprises were described by patients). A lack of examples of patient harm should be taken as an absence of evidence, not evidence of absence.^3^

Many practices were not able to easily identify patients who used their online access. Registration for online services is a blunt tool since registration does not entail use. Furthermore, registration is necessary for other linked services (for example, appointment booking and repeat prescription ordering) that may be the main reason for patients registering.^16^ This may explain why there were four participating practices in which no patients who responded to the invitation to take part had used online access to medical records, despite being registered for online services. Future research must carefully consider, in discussion with practices, how to identify patients using online access.

Patients who agreed to participate were mostly middle-aged and all were White British. More generally, the 2020 GP Patient Survey found that older people were less likely to use online services.^16^ Findings should be interpreted considering these limitations. Invitations to participate were in English, sent by post by GP practices, and required individuals to respond to the university researcher, which may have introduced sociocultural barriers for some communities. Future research could recruit in collaboration with community groups rather than GP practices to improve recruitment diversity, although it would be essential to ensure reciprocal benefits to avoid gatekeeper fatigue.

### Comparison with existing literature

Studies of online access to medical records have found patients have used them to understand their health and take greater responsibility for their care,^18^^,^^21^^–^24 and found the technology convenient and useful. This study’s findings, however, provide a more nuanced understanding.

Previous research has highlighted potential unintended consequences of online access. There are well known concerns around confidentiality and risk of patient coercion,^3^^,^^30^ patient confusion and anxiety,^3^^,^^6^^,^^31^ as well as the creation of additional clinician workload^19^ and widening of health inequalities.^32^^,^^33^ These are often hypothetical concerns, with unclear evidence they are realised in practice.^3^ This research supports the findings of previous studies examining hypothetical impacts, showing how the potential benefits and concerns about unintended consequences of online access are realised in practice.

### Implications for practice

Box 1 outlines the unintended consequences identified and offers mitigation guidance for clinicians and practice managers.

**Box 1.** Patient online access to health records guidance for clinicians and practice managers

**Potential unintended consequences**	**Mitigation**
Distress for patients at discovering unknown information in their record. Additional workload for staff supporting patients to prepare them for what to expect from online access.	Offer consultation to patients who you are concerned may be surprised or distressed by parts of their record. It may also be prudent to verbally summarise what is being documented during consultations to reduce the risk of future surprises or distress.
Non-patient-friendly information in records. For example, jargon, abbreviations, spelling mistakes, lack of context leading to offence, misinterpretation, or misunderstanding.	Where possible, clinicians should aim to reduce the use of jargon that may offend or confuse. However, it is imperative that the content of patient notes are kept as accurate as possible. Improving understandability for the patient must not compromise a high quality of care. Use texting or email to communicate reliable websites for information about conditions and treatments.
Clinician hesitance to document speculative concerns, diagnoses, or third-party information in records that are shared with patients.	Clinicians should continue their objective and factual documentation practices.
Additional workload to redact sensitive or third-party content.	Software to automate these tasks is available, but manual checking may still be required. It may be practical to have a staged rollout of online access, as patients individually request it, to spread the workload over a longer period of time.
Additional workload from managing more complex situations, for example, parent/teenager access, or individuals experiencing domestic violence and abuse.	It is recommended that complex situations are dealt with on a case-by-case basis before online access is provided. Where possible, this should involve a multidisciplinary team. In the case of parent/teenager access, depending on the competence and capacity of the young person, consent must be gained before releasing notes to parents.
Additional workload from patients querying/challenging notes and correcting errors.	Corrections or qualifications should be welcomed. Practices should expect that a minority of patients will present with questions regarding the content of the notes.

All the measures identified in Box 1 require extra work on behalf of practices and there is a clear up-front cost to preparing records and supporting patients to access their records. The accumulation of individually minor tasks may pose a more significant challenge as online access is scaled up; however, some workload may diminish over time. For example, it is a one-time task to redact sensitive content or correct an error highlighted by a patient. Providing access as patients individually request it spreads this workload over a longer period, making it potentially easier to absorb.

Making records accessible to patients raises questions about how they can best serve both clinicians and patients, when traditionally the primary purpose of medical records has been to support clinicians in the care of patients.^13^ Although issues around documentation practices are not new, patient online access being the ‘default position’^12^ throws this into sharper relief. The examples raised around access and others viewing the patient record (for example, a child’s parents or a coercive partner in issues of domestic violence) both highlight how patients’ relationships to others leads to unintended consequences when they do not fit the imagined picture of a patient exclusively accessing their record for their own use.

The unintended consequences described by both staff and patients show that, to achieve the intended consequences set out in NHS policy, practices face additional work that is necessary to prepare records for sharing and prepare patients about what to expect. It is crucial that practices are adequately supported and resourced to manage the unintended consequences of online access now that it is the default position.^12^
